# High admission glucose levels predict worse short-term clinical outcome in non-diabetic patients with acute myocardial infraction: a retrospective observational study

**DOI:** 10.1186/s12872-019-1140-1

**Published:** 2019-07-04

**Authors:** Xiao Song Ding, Shan Shan Wu, Hui Chen, Xue Qiao Zhao, Hong Wei Li

**Affiliations:** 1 Department of Cardiology, Cardiovascular Center, Beijing Friendship Hospital, Capital Medical University, 95 Yong An Road, Xi Cheng District, Beijing, 100050 People’s Republic of China; 2 National Clinical Research Center of Digestive Diseases, Beijing Friendship Hospital, Capital Medical University, 95 Yong An Road, Xi Cheng District, Beijing, 100050 People’s Republic of China; 3 Clinical Atherosclerosis Research Lab, Division of Cardiology, University of Washington, 1959 NE Pacific Street, Seattle, WA USA; 4 Department of Internal Medicine, Medical Health Center, Beijing Friendship Hospital, Capital Medical University, 95 Yong An Road, Xi Cheng District, Beijing, 100050 People’s Republic of China; 5 Beijing Key Laboratory of Metabolic Disorder Related Cardiovascular Disease, Beijing, 100069 People’s Republic of China

**Keywords:** Acute myocardial infarction, Admission hyperglycemia, Non-diabetes mellitus, In-hospital mortality

## Abstract

**Background:**

Patients with acute myocardial infarction (AMI) often accompanied by admission hyperglycemia, which usually predicts a poor clinical outcomes for non-diabetes mellitus. Appropriate cut-point to identify high risk individuals in these patients remains controversial.

**Methods:**

One thousand six hundred ninety-eight non-diabetes AMI patients in this retrospective study were divided into 3 groups according to admission glucose levels (euglycemia group≤140 mg/dL, moderate hyperglycemia group 141–179 mg/dL, severe hyperglycemia group≥180 mg/dL). The primary endpoint of this study was all-cause in-hospital mortality rate. In-hospital motality related risk factors was analyzed by multivariate binary logistic regression analyses.

**Results:**

All myocardial necrosis markers and Log NT-proBNP in severe hyperglycemia group were significantly higher than those in the other 2 groups. Logistic regression showed that independent predictors of the in-hospital mortality rate in non-diabetic patients with AMI were age (OR = 1.057, 95% CI 1.024–1.091, *P* < 0.001), logarithm of the N-terminal pro-brain natriuretic peptide (OR = 7.697, 95% CI 3.810–15.550, *P* < 0.001), insufficient myocardial reperfusion (OR = 7.654, 95% CI 2.109–27.779, *P* < 0.001), percutaneous coronary intervention (OR = 0.221, 95% CI 0.108–0.452, *P* < 0.001) and admission glucose (as categorical variable). Patients with moderate hyperglycemia (OR = 1.186, 95% CI 0.585–2.408, *P* = .636) and severe hyperglycemia (OR = 4.595, 95% CI 1.942–10.873, *P* = 0.001) had a higher all-cause in-hospital mortality rate compared with those with euglycemia after AMI in non-diabetic patients.

**Conclusions:**

The all-cause in-hospital mortality risk increases remarkably as admission glucose levels elevated in non-diabetic patients with AMI, especially in patients with admission glucose levels ≥180 mg/dL. Severe admission hyperglycemia could be regarded as prospective high-risk marker for non-diabetic AMI patients.

## Background

Many studies have shown that admission hyperglycemia (AHG) is common in patients with acute coronary syndrome (AMI) [1] and is also a risk factor for in-hospital death and complications [2]. Previous epidemiological studies have shown that approximately 25 to 50% of patients with AMI have co-existing hyperglycemia [3]. Recent studies have suggested that the effect of hyperglycemia on the outcomes of patients with AMI differs between patients with diabetes and those with previously undiagnosed diabetes [4]. The risks of cardiovascular events are higher in patients with acute myocardial infarction (AMI) who have not been diagnosed with diabetes mellitus (DM) or whose DM is diagnosed only after admission than in patients with AMI and normal blood glucose levels. Therefore, hyperglycemia is more predictive of adverse events in non-diabetic patients with AMI than in in those with DM [5]. Previous studies have analyzed the effect of insulin treatment for controlling admission blood glucose levels for a reduction in recent adverse events in patients with AMI [6]. However, there is no clear conclusion on which level of blood glucose control can benefit non-diabetic patients. In this retrospective study, we compared baseline data and the incidence of adverse events during hospitalization in non-diabetic patients with AMI with different admission blood glucose levels to determine possible influential factors.

## Methods

### Study population

**Fig. 1:**
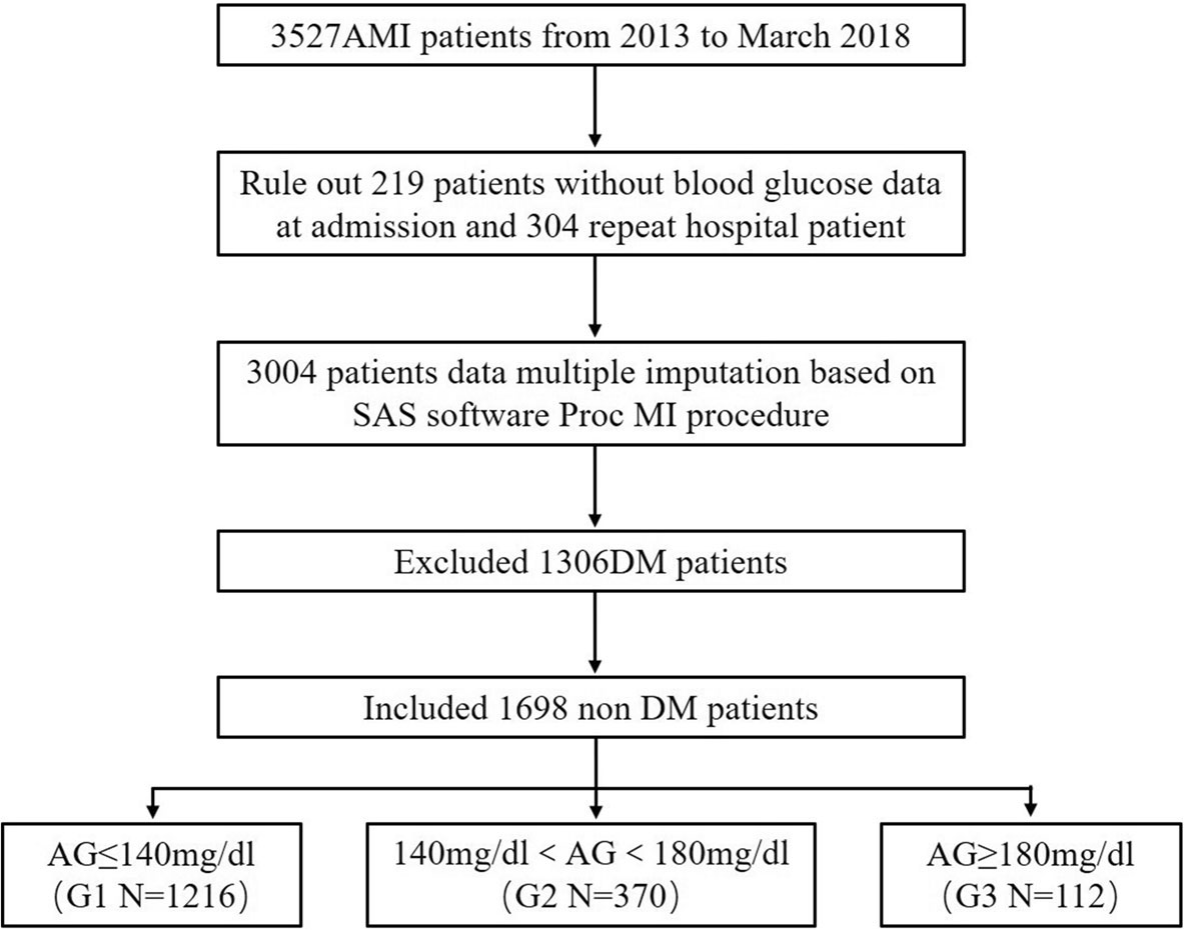
Patients screening flow chart. Acute myocardium infarction (AMI), Diabetes mellitus (DM), Admission glucose (AG)

This retrospective study was based on data from the Cardiovascular Center of Beijing Friendship Hospital Data Bank (CBD BANK). The study protocol was approved by the Beijing Friendship Hospital ethics committee (certification number: 2018-P2–051-01). A total of 3527 patients who were diagnosed with AMI (including ST-segment elevation myocardial infarction [STEMI] and non-ST-segment elevation myocardial infarction [NSTEMI]) during hospitalization in our hospital from January 2013 to March 2018 were identified by a search strategy. A total of 219 patients without blood glucose data at admission and 304 repeat hospital patients and 1306 patients with DM were excluded. Finally, 1698 patients were included in the final analysis. The optimal medical therapy for AMI was administered in every patient during hospitalization, according to the current guidelines. They were treated with dura antiplatelet drugs (aspirin plus clopidogrel/ticagrelor), ACEI, β blocker, statin were used as secondary prevention unless there were contraindications. Coronary angiography and percutaneous coronary intervention were performed if the doctors believe patients could benefit from the opration. These patients were divided into 3 groups according to the admission glucose (the first blood test analysis on admission) level as follows: euglycemia group, admission glucose levels ≤140 mg/dL (group 1, *n* = 1216); moderate hyperglycemia group, admission glucose levels > 140 and < 180 mg/dL (group 2, *n* = 370); and severe hyperglycemia group, admission glucose levels ≥180 mg/dL (group 3, *n* = 112). A flow chart of the cohort is shown in Fig. 1.

### Data collection and definitions

All individuals had a 12-lead electrocardiogram, transthoracic echocardiography, and laboratory tests (complete blood count, electrolytes, creatinine, lipid profile, glucose, and markers of myocardial necrosis) performed. The results of coronary angiography, percutaneous coronary intervention (PCI), and qualification for further treatment (conservative or invasive), as well as clinical event data during hospitalization, were collected. All of the eligible patients who were enrolled met the Third Universal Definition of Myocardial Infarction for AMI [7] as follows: detection of a rise and/fall on cardiac biomarker values (preferably cardiac troponin [cTn]) with at least one value above the 99th percentile upper reference limit and with at least one of the following factors. These factors were symptoms of ischemia, new or presumed new significant ST-segment-T wave changes or new left bundle branch block, development of pathological Q waves in an electrocardiogram, and imaging evidence of new loss of viable myocardium or new regional wall motion abnormality. Patients with DM were excluded on the basis of American Diabetes Association criteria as follows [8]: a history of diabetes; or the diagnosis of DM was made during hospitalization on the basis of an oral glucose tolerance test, fasting plasma glucose levels ≥126 mg/dL, 2-h plasma glucose levels ≥200 mg/dL, or glycosylated hemoglobin (HbA1c) ≥6.5%.

### Study endpoints

The primary endpoint of this study was the all-cause in-hospital mortality rate. In-hospital mortality was defined as death caused by all causes, including cardiac and non-cardiac death. Complications during hospitalization were defined as cardiogenic shock, stroke (including cerebral infarction and cerebral hemorrhage), fatal rapid arrhythmia (including ventricular tachycardia and ventricular fibrillation), and atrioventricular block (including second- and third-grade atrioventricular block) during hospitalization. The MACEs (major adverse cardiovascular events) were considered as secondary composite endpoint including: cardiovascular mortality, cardiogenic shock, ischemic stroke, fatal rapid arrhythmia and atrioventricular block.

### Statistical analysis

To avoid decreased statistical power and bias caused by the direct rule-out of missing data, we used multiple imputations before excluding patients with DM using the SAS software version 9.0 Proc MI procedure (SAS Institute, Cary, NC, USA) to account for missing data. The data were analyzed by SPSS software, version 20.0 (SPSS, Chicago, IL). Continuous variables are expressed as the median (P25, P75) and were compared by using the non-parametric rank sum test. Because none of these variables were normally distributed. Because N-terminal pro-brain natriuretic peptide (NT-proBNP) was extremely skewed distribution, it was log-transformed into a new variable log NT-proBNP. Categorical data are expressed by rates and proportions, and were analyzed by chi-square analysis. The relationship between admission glucose categories with in-hospital motality and other events was analyzed by using multivariate binary logistic regression analyses. First, univariate analysis of the following variables was performed: age, gender, age older than 75 years, left ventricular ejection fraction < 0.4, STEMI, percutaneous coronary intervention, insufficient myocardial reperfusion (postoperative blood flow< Thrombolysis in Myocardial Infarction TIMI 3), history of myocardial infarction, hypertension, hyperlipidemia, history of chronic kidney disease, history of cerebrovascular disease, current smoking, systolic blood pressure at admission, fasting plasma glucose levels on the day after admission, admission glucose levels, HbA1c, high-sensitivity C-reactive protein levels, total cholesterol levels, low-density lipoprotein cholesterol levels, creatinine levels, estimated glomerular filtration rate (eGFR), peak creatine kinase-MB (CK-MB) value, peak cardiac troponin I value, peak myoglobin (MYO) value, and left ventricular ejection fraction. Five variables including age, log NT-proBNP, PCI, Insufficient myocardium reperfusion and admission glucose were included in the final regression model. Variables with a *P* value < 0.05 were then included in the logistic regression equation to analyze the risk factors associated with all-cause in-hospital mortality. All analyses were 2-tailed and a *P* value < 0.05 was considered statistically significant. Adjusted *P* values (α = 0.05/3 = 0.016) were used in the pairwise comparison of in-hospital motality and other adverse events (Cardiac Motality, Cardiac Shock, Tachycardia, AVB) among the three groups.

## Results

Baseline data of continuous and categorical variables among the groups after being categorized by admission blood glucose levels are shown in Table 1. The severe hyperglycemia group had higher fasting blood glucose (*P* < 0.001) and HbA1c values (*P* < 0.001). The mean admission glucose value of the 12 patients died in group 3 was 228.76 mg/dl, maximum value 311.58 mg/dl, minimal value 182.34 mg/dl and SD 41.64 mg/dl (data not show in Table 1). All myocardial necrosis markers and Log NT-proBNP in severe hyperglycemia group were significantly higher than those in the other 2 groups. CK-MB(*P* < 0.001), cardiac troponin I(*P* < 0.001), MYO(*P* < 0.001) Log NT-proBNP was also significantly higher in severe hyperglycemia group than in the other 2 groups, which corresponded to a lower ejection fraction. For admission medications, there were no significant difference between three groups (Table 1).

**Table 1:** Baseline characteristics of non-DM patients stratified according to admission glucose

	AG ≤ 140 mg/dL M (P25, P75) *N* = 1216	140 mg/dL<AG<180 mg/dL M (P25, P75) *N* = 370	AG ≥ 180 mg/dL M (P25, P75) *N* = 112	*P*
Age (years)	63 (55, 76)	64 (57, 76)	66 (57, 73)	0.022
SBP (mmHg)	127 (114, 141)	124.00 (109.75, 138.00)	125.5 (110.00, 143.75)	0.008
FPG (mg/dL)	91.08 (83.16, 101.11)	101.7 (91.26, 114.71)	109.17 (92.88, 136.80)	<0.001
AG (mg/dL)	114.12 (103.36, 125.77)	153.36 (146.11, 163.8)	204.75 (190.21, 229.63)	<0.001
HbA1C(%)	5.60 (5.3, 5.8)	5.70 (5.4, 6.0)	5.80 (5.60, 6.20)	<0.001
hs-CRP (ng/ml)	6.77 (2.25, 15.26)	7.47 (2.58, 15.95)	8.93 (2.64, 21.21)	0.201
TC (mmol/L)	4.34 (3.72, 5.08)	4.39 (3.75, 4.99)	4.23 (3.58, 4.81)	0.363
LDL-C (mmol/L)	2.51 (2.02, 3.04)	2.50 (2.06, 2.97)	2.44 (1.93, 2.87)	0.250
Cr (mg/dL)	0.95 (0.84, 1.09)	1.05 (0.83, 1.08)	0.98 (0.84, 1.20)	0.228
eGFR (ml/min·1.73m^2^)	80.70 (64.52, 94.89)	77.26 (65.51, 93.53)	76.51 (60.17, 87.93)	0.094
log NT-proBNP	3.16 (2.68, 3.66)	3.27 (2.78, 3.80)	3.33 (2.99, 3.87)	<0.001
CK-MB ( ng/ml)	32.10 (4.5, 105.16)	59.89 ( 7.9, 165.75)	74.90 ( 17.35, 209.25)	<0.001
cTnI ( ng/ml)	3.31 (0.53, 13.23)	6.21 (0.56, 20.17)	5.95 (1.18, 33.28)	<0.001
MYO (ng/ml)	101.5 (35.6, 250.94)	150.50 (49.83, 301.36)	175.39 (82.78, 428.25)	<0.001
EF	0.61 (0.54, 0.66)	0.59 (0.51, 0.66)	0.58 (0.51, 0.64)	0.003
HS (day)	8.0 (6.0, 10.0)	8.0 (6.0, 11.0)	8.0 (6.0, 10.0)	0.149
Male	930 (76.5)	269 (72.7)	130 (76.8)	0.32
Age over 75	324 (26.6)	110 (29.7)	25 (22.3)	0.25
EF below 0.4	88 (7.2)	27 (7.3)	7 (6.2)	0.92
STEMI	585 (48.1)	229 (61.9)	75 (67.0)	<0.001
PCI	883 (72.6)	285 (77.0)	89 (79.5)	0.09
IMR	14 (1.2)	15 (4.1)	6 (5.4)	<0.001
OMI	130 (10.7)	43 (11.6)	11 (9.8)	0.82
Hypertension	7718 (59.0)	241 (65.1)	72 (64.3)	0.08
Dyslipidemia	511 (42.0)	170 (45.9)	52 (46.4)	0.32
CKD	55 (4.5)	19 (5.1)	5 (4.5)	0.88
Stroke history	167 (13.7)	65 (17.6)	14 (12.5)	0.15
Current smoking	589 (48.4)	159 (43.0)	48 (42.9)	0.12
Medication befor admission				
Aspirin	134 (11)	33 (8.9)	16 (14.3)	0.24
CCB	325 (26.7)	115 (31.1)	37 (33.0)	0.13
β-blocker	131 (10.8)	48 (13.0)	13 (11.6)	0.50
ACEI	104 (8.6)	36 (9.7)	9 (8.0)	0.75
ARB	177 (14.6)	44 (11.9)	19 (17)	0.29
Statin	136 (11.2)	56 (15)	10 (8.9)	0.07

*AG* Admission glucose, *SBP* Systolic blood pressure, *FPG* Fasting plasma glucose, *HbA1c* Glycosylated hemoglobin, *hs-CRP* High-sensitivity C-reactive protein, *TC* Total cholesterol, *LDL-C* Low-density lipoprotein cholesterol, *Cr* Creatinine, *eGFR* estimated glomerular filtration rate, *NT-proBNP* N-terminal pro-brain natriuretic peptide, *CK-MB* Creatine kinase-MB, *cTnI* Cardiac troponin I, *MYO* Myoglobin, *EF* Ejection fraction, *HS* Hospital stay, *STEMI* ST-segment elevation myocardial infraction, *PCI* Percutaneous coronary intervention, *IMR* Insufficient myocardium reperfusion, *OMI* Old myocardial infraction, *CKD* Chronic Kidney Disease, *CCB* Calcium channel blocker, *ACEI* Angiotensin-converting enzyme inhibitor, *ARB* Angiotensin receptor blocker

**Fig. 2:**
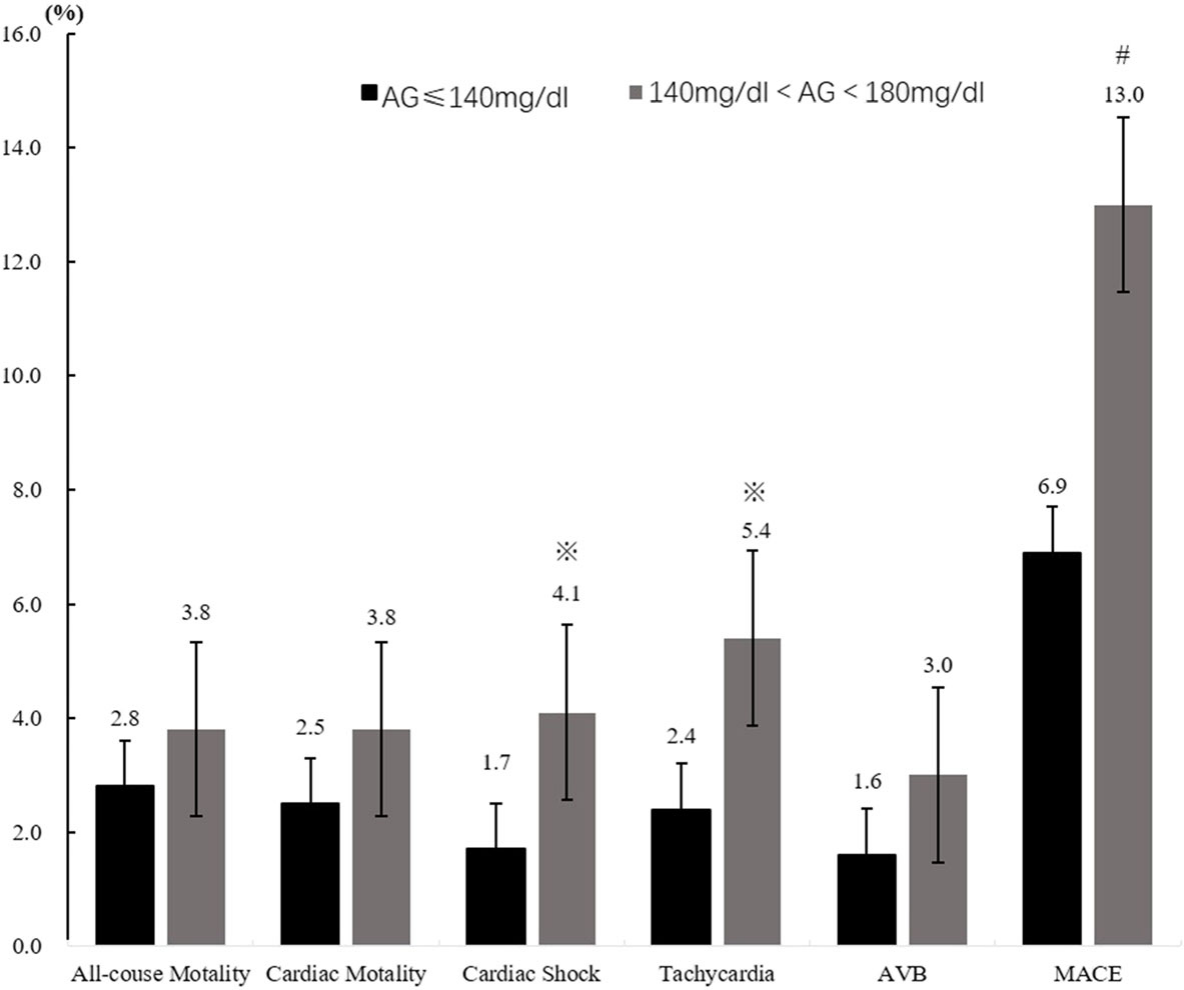
All-cause mortality and complications comparison between euglycemia group and moderate hyperglycemia groups. ※*P* < 0.01, #*P* < 0.001, AVB Atrioventricular block, MACE Major adverse cardiovascular events

**Fig. 3:**
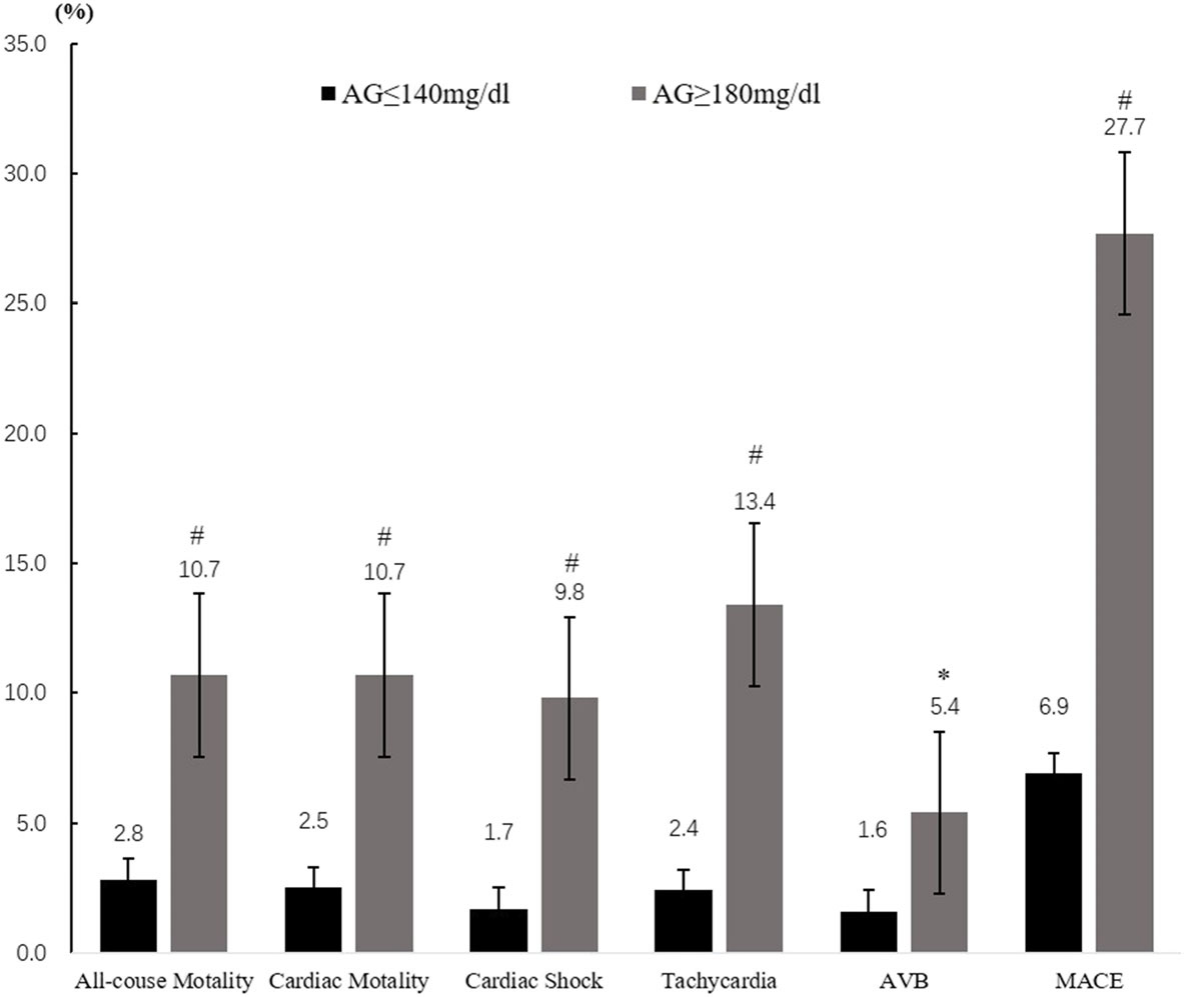
All-cause mortality and complications comparison between euglycemia group and severe hyperglycemia. *P < 0.05, #*P* < 0.001, AVB Atrioventricular block, MACE Major adverse cardiovascular events

**Fig. 4:**
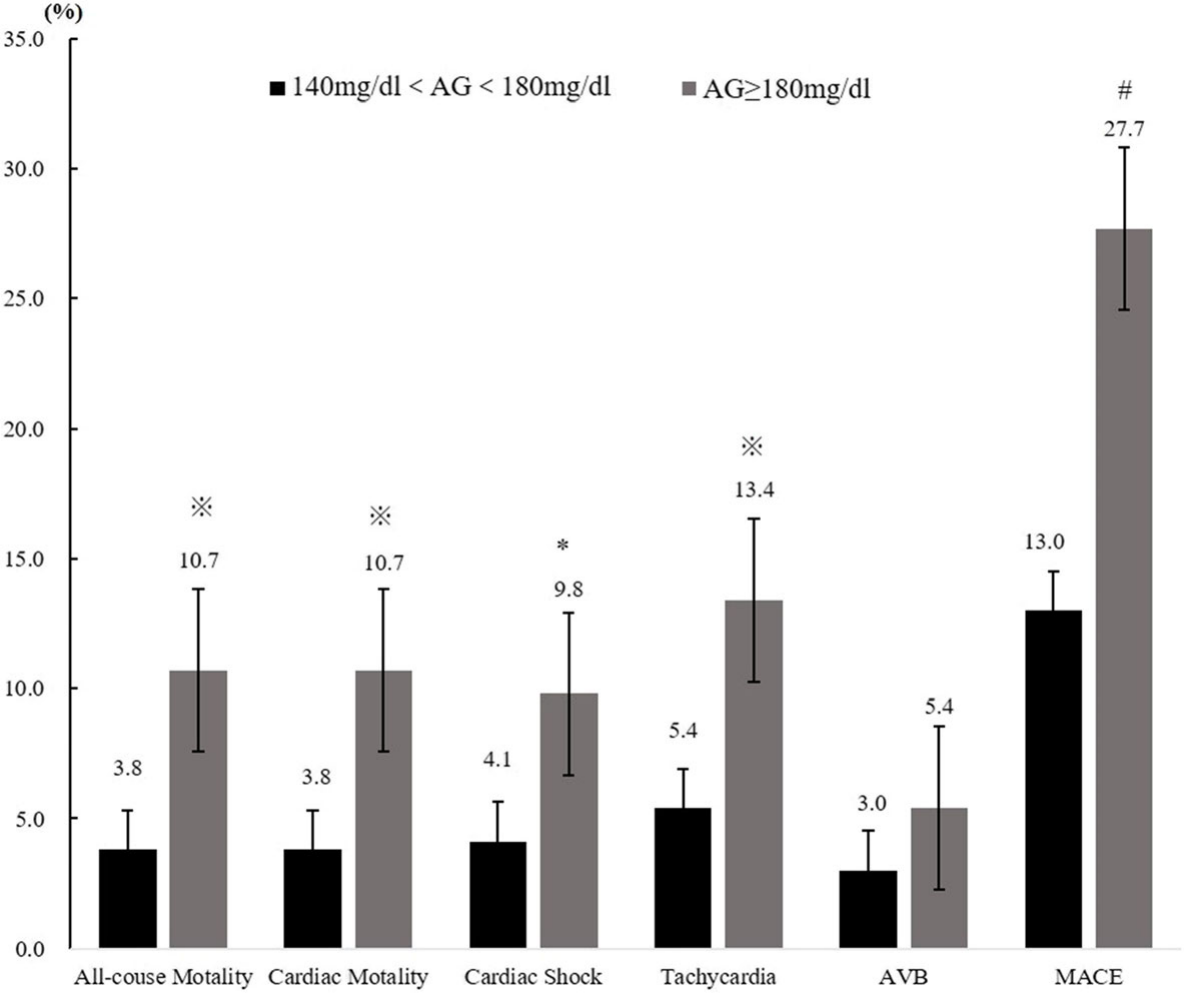
All-cause mortality and complications comparison between moderate hyperglycemia group and severe hyperglycemia. *p < 0.05, ※*P* < 0.01, #*P* < 0.001, AVB Atrioventricular block, MACE Major adverse cardiovascular events

The in-hospital all-cause mortality rate and cardiac mortality rate significantly elevated as admission blood glucose levels increased. Other adverse events rates gradually increased with admission blood glucose categories grade up and differences of total MACE events between any 2 of the 3 groups were statistically significant. There were significant differences in all-cause and cardiac motality between groups 2 and 3, not between group 1 and 2 (Figs. 2, 3 and 4).

Logistic regression analysis showed that admission glucose was one of the independent predictors of the in-hospital mortality. The in-hospital all-cause motality risk comparisons between groups 3 and 1 and groups 3 and 2 were statistical significant (OR = 4.595, P<0.001 and 3.873, *P* = 0.006 respectively, Table 2).

**Table 2:** Binary logistic regression analysis of in-hospital all-cause mortality-related factors

	OR	95% C.I.		*P*
Age	1.057	1.024	1.091	0.001
Log NT-proBNP	7.697	3.810	15.550	<0.001
PCI	.221	.108	.452	<0.001
IMR	7.654	2.109	27.779	0.002
AG (VS Group1)				
Group2	1.186	.585	2.408	0.636
group3	4.595	1.942	10.873	0.001
AG (VS Group2)				
Group3	3.873	1.485	10.100	0.006

*OR* Odds ratio, *C.I.* Confidence interval, *Log NT-proBNP* Logarithm of the N-terminal pro-brain natriuretic peptide, *PCI* Percutaneous coronary intervention, *IMR* Insufficient Myocardium Reperfusion, *AG* Admission glucose, Group 1 admission blood glucose ≤140 mg/dL, group 2 admission blood glucose > 140 and < 180 mg/dL, group 3 admission blood glucose ≥180 mg/dL

## Discussion

In the current study, we analyzed in-hospital mortality rate and other comorbidities of AMI patients without DM grouped by two admission blood glucose cut-point value (140 mg/dL and 180 mg/dL). We found that, patients with moderate hyperglycemia and severe hyperglycemia had higher in-hospital mortality rates after AMI compared with patients with euglycemia patients. MACE events rise up parallelly with admission glucose upgrade. Logistic regression analysis showed that admission glucose was a independent predictors of the in-hospital mortality rate in non-diabetic AMI patients. In-hospital motality risk of the severe hyperglycemia was 3.873 folds (*P* = 0.006) than moderate admission hyperglycemia group.

Several decades ago, AHG was recognized as a common phenomenon in patients with AMI. The incidence of AHG ranges from 51 to 61.7% (with a cut-off value of 140 mg/dL) [3]. Increased blood glucose levels in patients with AMI have been described as AHG, high admission blood glucose levels, acute hyperglycemia, and stress hyperglycemia [9]. The American Heart Association Diabetes Committee defined AMI accompanied by stress hyperglycemia as an admission plasma glucose level of > 140 mg/dL in its scientific statement [10]. However, AHG cut-off values differ in different studies. The American Diabetes Association recommends that patients with severe disease have blood glucose levels > 180 mg/dL and need to start insulin treatment, and the recommended target glucose range is from 140 to 180 mg/dL [11]. Therefore, in this study, we set the grouping cut-points as 140 mg/dL and 180 mg/dL.

Some clinicians consider that AHG is only a criterion reflecting severity of AMI because it is common in patients who are older, diabetic, and have STEMI with acute heart failure [12]. However, the definite relationship between AHG and short and long-term poor prognoses shows that AHG is also a factor that contributes to progression of disease [2].

AHG often predicts a poor short- or long-term prognosis in patients with AMI. The in-hospital mortality rate was 3.6 times higher and the 1- to 3-year mortality rate was 2.26 times higher in patients with AMI and AHG than in those without AHG [5] There are several explanations for hyperglycemia in patients with AMI. Previous studies have shown that catecholamine and steroid hormone levels are significantly increased and positively correlated with stress levels in patients with AMI [13, 14]. Additionally, a stress-induced elevation in catecholamine levels can inhibit pancreatic β-cell function and thus cause a decline in insulin levels [15]. Whether this hyperglycemic response is associated with more myocardial infarction events, with impaired myocardial function, or both remains unclear.

In a previous study, in patients with AHG, the proportion of low TIMI flow scores was higher than that in patients with normal blood glucose levels after PCI [16]. Infarct size was larger [17], and the incidence rates of malignant arrhythmia [18] and cardiac shock [19] were also higher in patients with AHG, especially in non-DM patients than in those without AHG [16]. In the current study, CK-MB, cardiac troponin I, and MYO levels were higher in the severe hyperglycemia group of non-DM patients with AMI. This finding suggested that myocardial injury was in parallel with admission blood glucose levels. This result is accordance with previous study [3]. There are several possible mechanisms to explain the association between AHG and AMI adverse events. Hyperglycemia leads to thrombogenesis by activating platelets and affects fibrinolysis [20, 21]. Hyperglycemia is associated with the no-reflow phenomenon [22]. Furthermore, other factors, including oxidative stress [23], insulin resistance, and massive catecholamine production increase free fatty acid levels, and excessively high free fatty acid levels have toxic effects on infarcted and ischemic myocardium [15]. Another potential mechanism may include endothelium disfunction that occurs in patients with admission hyperglycemia [24].

Many studies have reported that AMI accompanied by AHG is an independent predictor of heart failure and death [19]. These clinical outcomes are even worse in non-diabetes patients [5]. A study on the relationship between AHG and left ventricular function showed that patients with acute AHG before revascularization had a lower postoperative left ventricular ejection fraction than did those without AHG [3]. Furthermore, those patients had a significantly lower left ventricular ejection fraction before discharge and significantly less improvement in left ventricular function. This study suggested that acute AHG can impair left ventricular function, even after successful revascularization. In our study, we found that patients with high admission blood glucose levels were more likely to have poor coronary artery flow and a lower ejection fraction after PCI, which is consistent with previous studies [25].

Most previous studies on the clinical outcomes of patients with AHG and AMI were conducted in Western countries, but a few studies examined these outcomes in Asian populations. Several Chinese studies have investigated the relationships between blood glucose levels at admission and clinical outcomes. In a preliminary study in our center, Song et al. concluded that AHG should be regarded as a strong risk factor for in-hospital and 2-year all-cause mortality in patients with AMI [26]. Li et al. also found that the in-hospital mortality rate began to rise in patients with non-DM and blood glucose levels of 162 mg/dl or higher [27]. Another study that focused on the prognosis of Chinese patients with non-DM and AMI showed that the 7- and 30-day mortality rates gradually increased with increasing blood glucose levels at admission, and elevated HbA1c levels showed that AHG was an independent predictor of in-hospital mortality, but elevated HbA1c levels were not [28]. Logistic regression analysis in our study also showed that admission blood glucose levels ≥180 mg/dL were an independent predictive factor associated with in-hospital mortality, except for the variates of age, log NT-proBNP, PCI treatment, and insufficient myocardial reperfusion.

Patients with AMI and undiagnosed diabetes, even if their blood glucose levels are elevated on admission, do not receive sufficient attention and adequate medical treatment in most cases. Early positive glycemic therapy is thought to improve the clinical outcome, but several clinical studies have shown that mortality rates do not improve [29]. This finding may be related to failure of controlling blood glucose levels according to the protocol or a stratified analysis strategy was not used for diabetic and non-diabetic patients [30]. Our study suggests that blood glucose control of non-diabetic patients with 180 mg/dL as the target of control may help to reduce the in-hospital mortality risk and adverse events. However, more prospective therapeutic clinical studies are required to confirm this conclusion.

### Study limitations

There are several limitations of this study. It’s a single-center observational study based on retrospective design, and the course of hyperglycemia was not all evaluated after the acute event, even some patients rechecked by OGTT test, that means some patients could have had undiagnosed DM or impaired glucose tolerance on admission and may have been included in our study group. The in-hospital management of hyperglycemia and used glycemic target choice were not included. Long-term patient follow-up should carried out, that would be helpful to define long term effects.

## Conclusion

Our study shows that the all-cause in-hospital mortality risk remarkably increases in non-diabetic patients with AMI and elevated blood glucose levels at admission, especially in patients with admission glucose levels ≥180 mg/dL. Severe admission glucose elevation could be a useful marker for identifying patients with a high mortality risk in non-diabetic AMI patients.

## Data Availability

The datasets used and analysed during the present study are available from the corresponding author on reasonable request.

## Abbreviations

AHG: Admission hyperglycemia
AMI: Acute myocardial infarction
AVB: Atrial ventricular block
CK-MB: Creatine kinase-MB
cTn: Cardiac troponin
DM: Diabetes mellitus
eGFR: Estimated glomerular filtration rate
HbA1c: Glycosylated hemoglobin
Log NT-proBNP: Logarithm of the N-terminal pro-brain natriuretic peptide
MACEs: Major adverse cardiovascular events
MYO: Myoglobin
NSTEMI: Non-ST-segment elevation myocardial infarction
PCI: Percutaneous coronary intervention
STEMI: ST-segment elevation myocardial infarction
TIMI: Thrombolysis in myocardial infarction
